# Marine natural products that inhibit osteoclastogenesis and promote osteoblast differentiation

**DOI:** 10.1007/s11418-022-01622-5

**Published:** 2022-04-10

**Authors:** Ahmed H. H. El-Desoky, Sachiko Tsukamoto

**Affiliations:** 1 Graduate School of Pharmaceutical Sciences, Kumamoto University, Kumamoto, 862-0973 Japan; 2 Pharmaceutical Industries Research Division, Pharmacognosy Department, National Research Centre, 33 El Bohouth St. (Former El Tahrir St.), Dokki, P.O. 12622, Giza, Egypt

**Keywords:** Marine organisms, Osteoporosis, Osteoclast, Osteoblast, RANKL, TRAP

## Abstract

Osteoporosis is a disease that affects the quality of life of elderly people. The balance between bone formation mediated by osteoblasts and bone resorption by osteoclasts is important to maintain the normal bone condition. Therefore, the promotion of osteoblast differentiation and the suppression of osteoclastogenesis are effective strategies for osteoporosis treatment. Marine organisms are a promising source of biologically active and structurally diverse secondary metabolites, and have been providing drug leads for the treatment of numerous diseases. We describe the marine-derived secondary metabolites that can inhibit receptor activator of nuclear factor-κB ligand (RANKL)-induced osteoclastogenesis and promote osteoblast differentiation.

## Introduction

In the European Union, the International Osteoporosis Foundation reported that 22 million women and 5.5 million men aged 50–84 had osteoporosis in 2010 [1]. In the United States, 53.6 million women and men over 50 years old had osteoporosis in 2010, which is considered to be a major public health threat [2]. In Japan, a nationwide survey of hip fractures, the most prominent sign of osteoporosis, is conducted every 5 years. This survey revealed that the total number of hip fractures was 193,400, consisting of 44,100 men and 149,300 women in 2017 [3]. Although the mortality rate is lower than that of other diseases, such as cancer and cardiovascular diseases, osteoporosis affects the quality of life of elderly people [4].

Several approaches have been adopted for the treatment of osteoporosis. Hormonal replacement therapy with estrogen has been applied for the prevention and treatment of osteoporosis [5]. However, risks of breast and endometrial cancers are increased with the prolonged use of estrogen [6]. Calcitonin is used for the suppression of bone resorption, but it has side effects, including flushing, nausea, and diarrhea [7]. Bisphosphonates are common for osteoporosis [8], but recently they were reported to lead to pathological conditions such as osteonecrosis of the jaw, atrial fibrillation, excessive suppression of bone turnover, hypocalcemia, and acute inflammatory response.

**Fig. 1:**
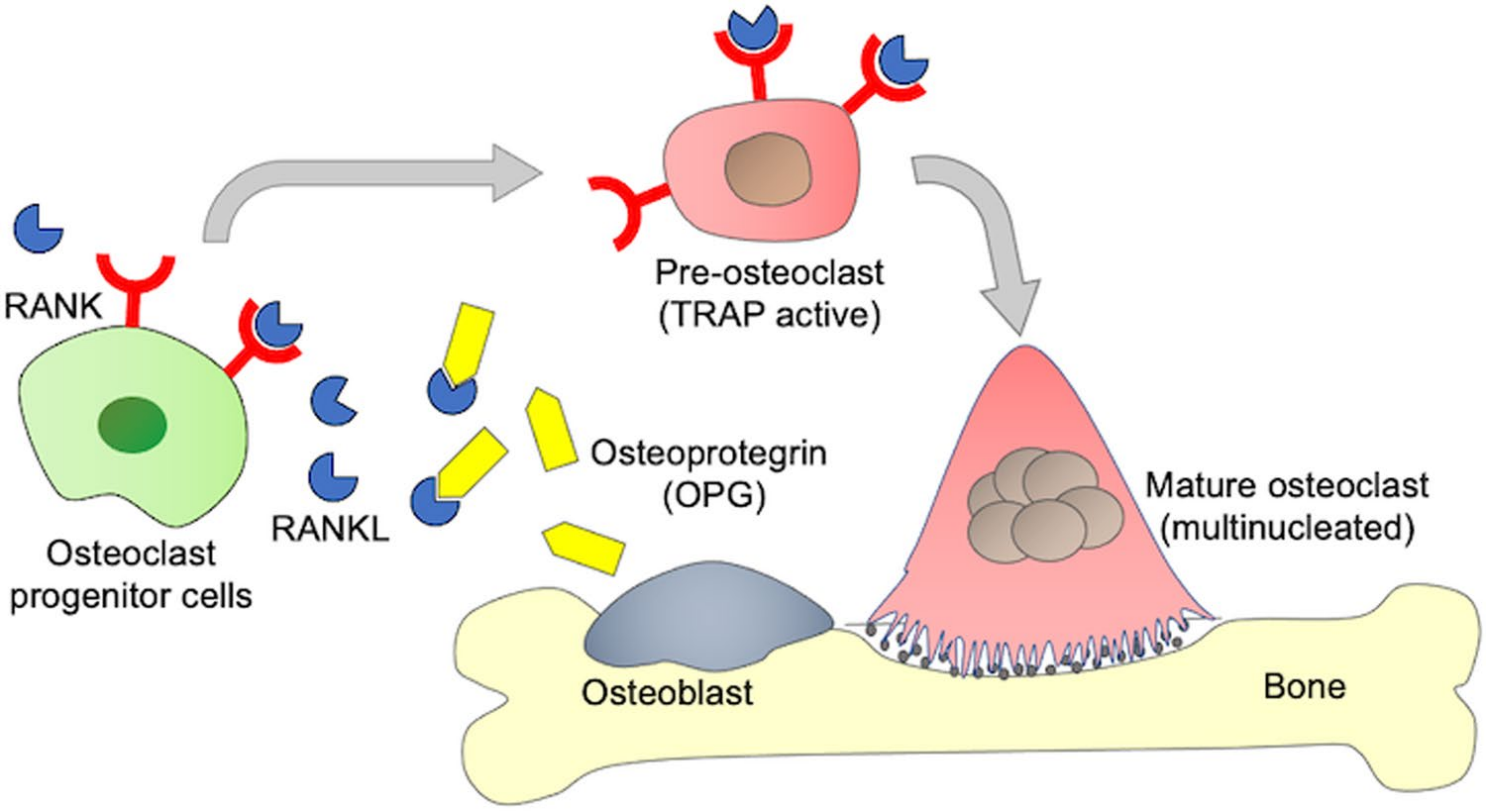
RANKL-induced osteoclastogenesis and bone resorption

The balance between bone formation by osteoblasts and bone resorption by osteoclasts is important to maintain the normal bone condition. Therefore, the suppression of bone resorption by osteoclasts is an effective strategy for the treatment of osteoporosis in addition to the promotion of osteoblast differentiation. The monocyte/macrophage lineage differentiates into osteoclasts by stimulation with receptor activator of nuclear factor-κB ligand (RANKL) (Fig. 1). On the other hand, osteoblasts produce a decoy receptor, osteoprotegerin (OPG), that binds to excess RANKL [9].

RANKL stimuli are known to activate several downstream signaling pathways such as nuclear factor-κB (NF-κB) and mitogen-activated protein kinases (MAPKs). The activation of these pathways upregulates the expression of osteoclast-specific genes, including those encoding tartrate-resistant acid phosphatase (TRAP) and other enzymes involved in cell fusion and acidification of the subcellular space. These processes lead to the degradation of bone matrix proteins and dissolution of bone minerals [10]. Therefore, the compounds that affect osteoclastogenesis have attracted much attention for the treatment of osteoclast-related diseases [11, 12].

Marine organisms exhibit wide diversity such as plants, microorganisms, and animals including sponges, cnidarians, bryozoans, mollusks, tunicates, and echinoderms. For decades, these marine invertebrates have been considered to be a potential source of unique drug leads with diverse structures that are unobtainable by combinatorial syntheses. Marine secondary metabolites have fascinating biological activities and have been used for numerous biological functions such as repelling predators, protection against infection, and communication among individuals of inter/intraspecies. This suggests that marine organisms have survived using their metabolites, thereby constructing the ecological system [13–15]. The marine environment varies in depth, current, temperature, light, and nutritional status, and produces extraordinary phenomena, such as hydrothermal deposit and effusion of poisonous gas, which may influence marine organisms to produce secondary metabolites with different structures and biological activities. Many reports revealed their high potential for medicinal, agrochemical, nutraceutical, and cosmetic uses [16]. We review the marine natural products that inhibit osteoclastogenesis and induce osteoblast differentiation for use as interventions to improve osteoporosis.

## Marine natural products that inhibit osteoclastogenesis

**Fig. 2:**
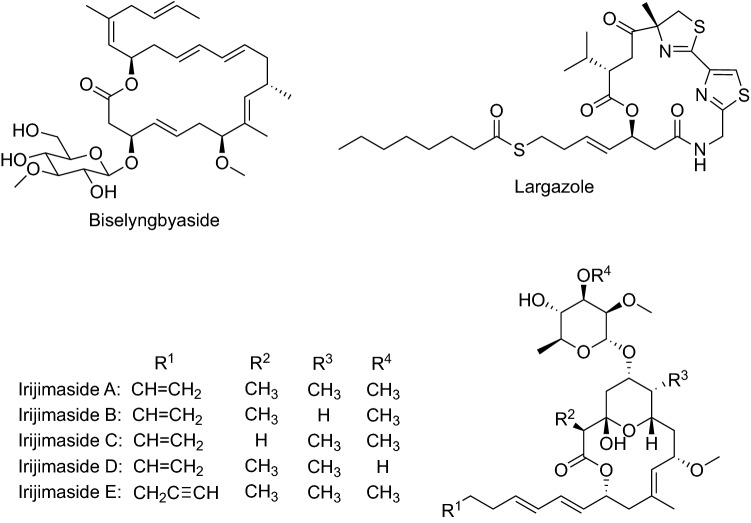
Structures of compounds that inhibited RANKL-induced osteoclastogenesis isolated from cyanobacteria

Biselyngbyaside is an 18-membered macrolide glycoside, first isolated from the marine cyanobacterium *Lyngbya* sp. collected from the reef of Bise, Okinawa, Japan, as a cytotoxic compound against a panel of human tumor cell lines (Fig. 2) [17]. Afterward, biselyngbyaside was demonstrated to inhibit RANKL-induced osteoclastogenesis in murine macrophages, RAW264 cells, and primary bone marrow-derived macrophages. It inhibited the activity of TRAP, which is an osteoclast-specific enzyme, in a dose-dependent manner with an IC_50_ value of 6 nM without cytotoxicity to RAW264 macrophages. Biselyngbyaside inhibited RANKL-induced osteoclast formation at 30 nM. In addition, it inhibited osteoblast-mediated osteoclast formation in co-cultures of osteoclasts with osteoblastic cells (UAMS-32) [18].

Largazole, a 16-membered cyclic depsipeptide, was isolated from a marine cyanobacterium *Symploca* sp. collected from Key Largo, Florida Keys, as an antiproliferative agent (Fig. 2) [19]. The same authors synthesized largazole, indicated to be a class I HDAC inhibitor [20], and reported antitumor activity in a xenograft mouse model [21]. Furthermore, it exhibited both in vitro and in vivo osteogenic activity mediated through the increased expression of Runx2 (a Runt protein) and bone morphogenetic proteins (BMPs) [22].

Irijimasides A–E, 14-membered macrolide glycosides, were isolated from a marine cyanobacterium *Okeania* sp. collected from Irijima, Okinawa, Japan (Fig. 2). These compounds dose-dependently inhibited RANKL-induced TRAP activity of RAW264 macrophage cells without significant cytotoxicity [23].

**Fig. 3:**
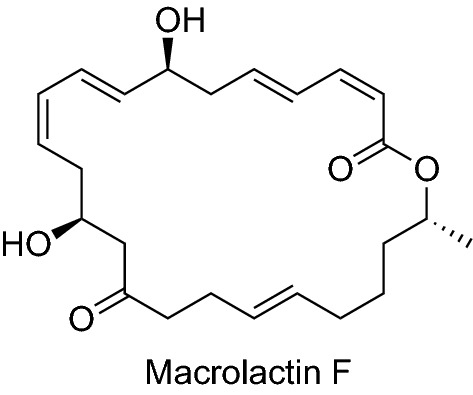
Structures of compounds that inhibited RANKL-induced osteoclastogenesis isolated from a bacterium

Macrolactins A–F, 24-membered ring lactones and related β-glucopyranosides, were isolated from an unidentified deep-sea marine bacterium collected in the North Pacific (Fig. 3). Macrolactin A exhibited antibacterial, antiviral, and cytotoxic activities [24]. Macrolactin F inhibited RANKL-induced osteoclastogenesis in primary bone marrow-derived macrophages (BMMs) by suppressing Akt, MAPK, and nuclear factor of activated T cells c1 (NFATc1) pathways. Moreover, the compound promoted osteoblastogenesis through a BMP-2/smad/Akt/Runx2 signaling pathway [25].

**Fig. 4:**
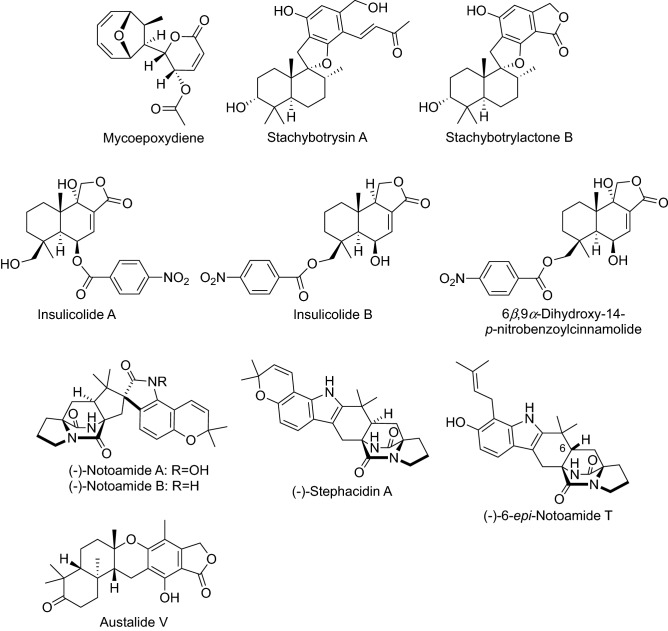
Structures of compounds that inhibited RANKL-induced osteoclastogenesis isolated from fungi

Mycoepoxydiene, an epoxycyclooctadiene-δ-1actone, was obtained from the solid-state fermentation of the fungus (OS-F66617) isolated from twig litter collected from a deciduous alluvial forest near Curitiba, Brazil (Fig. 4) [26]. Thereafter, mycoepoxydiene was reported to inhibit RANKL-induced osteoclast differentiation and to prevent bone loss in ovariectomized mice [27].

Stachybotrysin A and stachybotrylactone B, phenylspirodrimane derivatives, were isolated from the liquid cultures of the marine-derived fungus *Stachybotrys* sp. KCB13F013 collected from the sediment of Wi-island, South Korea (Fig. 4). Stachybotrysin A inhibited osteoclast differentiation in BMMs by suppressing RANKL-induced expression of the osteoclast-specific genes p-ERK, p-JNK, p-p38, c-Fos, and NFATc1 [28].

Insulicolide A is a benzoyl sesquiterpenoid containing a nitro group isolated from the marine fungus *Aspergillus insulicola* (Fig. 4) [29]. Insulicolide B and its derivatives were successively isolated from the marine-derived fungus *A. ochraceus* Jcma1F17 and demonstrated cytotoxicity [30]. The effects of the nitrobenzoyl sesquiterpenoids on osteoclastogenesis were examined, and 6β,9α-dihydroxy-14-*p*-nitrobenzoylcinnamolide exhibited the most potent suppression of RANKL-induced osteoclastogenesis and bone resorption at 0.5 µM [31].

Notoamides A–D are prenylated indole alkaloids isolated from the marine-derived fungus *Aspergillus protuberus* MF297-2 along with a known compound (+)-stephacidin A (Fig. 4) [32]. Later, two antipodes, (–)-stephacidin A and (+)-notoamide B, were isolated from the terrestrial fungi *A. amoenus* NRRL 35,600 [33]. Biological activities were assessed with natural and synthetic analogues, and a series of the (–)-enantiomers of notoamides A and B, 6-*epi*-notoamide T, and stephacidin A inhibited RANKL-induced osteoclastogenesis more strongly than their respective (+)-enantiomers [34]. Among the tested compounds, a synthetic compound (–)-6-*epi*-notoamide T was the most potent, with an IC_50_ value of 1.7 µM.

Meroterpenoids, austalides V − X, were isolated from the culture of the marine-derived fungus *Penicillium rudallense* as inhibitors of osteoclast differentiation (Fig. 4) [35]. Austalide V was the most potent inhibitor of RANKL-induced osteoclast differentiation, with an IC_50_ value of 1.9 μM.

**Fig. 5:**
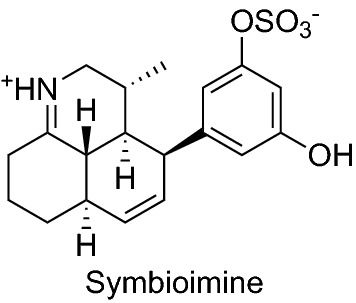
Structures of a compound that inhibited RANKL-induced osteoclastogenesis isolated from a dinoflagellate

Symbioimine is a unique amphoteric sulfated iminium compound isolated from the symbiotic marine dinoflagellate *Symbiodinium* sp. The compound inhibited RANKL-induced osteoclastogenesis in RAW264 cells with an IC_50_ value of 44 µg/mL without cytotoxicity (Fig. 5) [36].

**Fig. 6:**
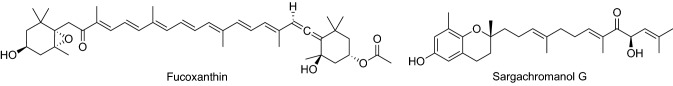
Structures of compounds that inhibited RANKL-induced osteoclastogenesis isolated from algae

Fucoxanthin is an oxygenated carotenoid contained in edible brown algae such as *Undaria pinnatifida* (wakame), *Laminaria japonica* (kombu), and *Eisenia bicyclis* (arame) (Fig. 6). Several health benefits of fucoxanthin have been reported such as suppression of adipocyte differentiation, antimutagenicity, and antiproliferative activity against cancers. Fucoxanthin inhibited RANKL-induced osteoclastogenesis in RAW264.7 macrophages at a concentration of 2.5 µM without cytotoxicity [37].

Sargachromanol G was first isolated from the brown alga *Sargassum siliquastrum* as antioxidant compounds with 15 new meroterpenoid congeners (Fig. 6) [38]. Later, the inhibitory effects of sargachromanol G on osteoclast formation from RANKL-treated RAW264.7 cells were reported, accompanied by suppression of the expression of osteoclast-specific markers such as *TRAP*, cathepsin K (*CTSK*), matrix metalloproteinase 9 (*MMP9*), and calcitonin receptor (*CTR*). Further examination of the mechanism of action revealed that sargachromanol G inhibited RANKL-induced activation of NF-κB by suppressing IκB-α degradation and inhibition of RANKL-induced phosphorylation of MAPKs (p38, JNK, and ERK) [39].

**Fig. 7:**
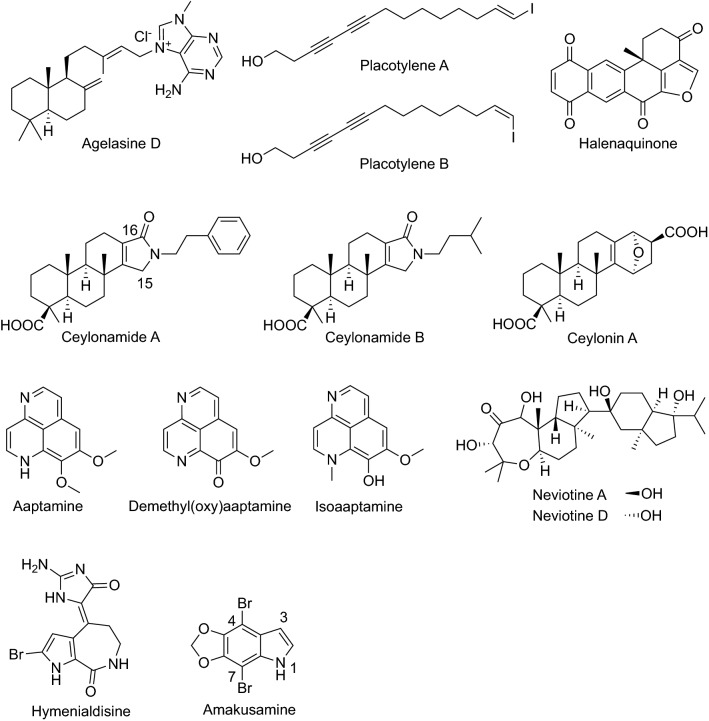
Structures of compounds that inhibited RANKL-induced osteoclastogenesis isolated from sponges

Agelasines were isolated from the marine sponge *Agelas nakamurai* Hoshino collected in Okinawa (Fig. 7) [40–42]*.* They were identified as monocyclic and bicyclic diterpenes with a 9-methyladeninium, and exhibited inhibitory effects against Na,K-ATPase. Later, agelasine D was reported to inhibit RANKL-induced osteoclastogenesis in BMMs through inhibiting the expression of osteoclastic markers, TRAP, cathepsin K, and MMP9. Furthermore, it suppressed RANKL-induced mRNA expression of dendritic cell-specific transmembrane protein (DC-STAMP) and osteoclast-stimulatory transmembrane protein (OC-STAMP). Moreover, RANKL-induced expression and protein production of both c-FOS and NFATc1 were downregulated by agelasine D [43].

Placotylenes A and B were isolated from a sponge *Placospongia* sp. collected in the South Sea, Korea and identified as unique iodinated linear polyacetylenes (Fig. 7). Placotylene A inhibited RANKL-induced osteoclastogenesis in BMMs at 10 µM, whereas its regioisomer placotylene B did not up to 100 µM. This inhibition was accompanied by the suppression of transcriptional and translational expression of NFATc1 [44].

During screening of osteoclastogenesis inhibitors from the extracts of marine sponges and marine-derived fungi, halenaquinone was isolated from the extract of the marine sponge *Petrosia alfiani* collected in Indonesia (Fig. 7). Halenaquinone inhibited RANKL-induced upregulation of TRAP activity of RAW264 cells, with an IC_50_ value of 2 µM. Studies on the inhibitory mechanism suggested that halenaquinone suppresses the NF-κB and Akt signaling pathways [45].

Ceylonamides A–F, nitrogenous spongian diterpenes, were isolated from the marine sponge *Spongia ceylonensis* collected in North Sulawesi, Indonesia (Fig. 7). Among these, ceylonamides A and B inhibited TRAP activity in RANKL-induced RAW264 cells, with an IC_50_ value of 13 and 18 μM, respectively, and formation of TRAP-positive multinuclear osteoclasts without cytotoxicity. Structure–activity relationship (SAR) studies revealed that the compounds having the amide carbonyl at C-16 inhibited more potently than those with the amide carbonyl at C-15. In addition, the presence of a bulkier substituent at the amide nitrogen resulted in more potent inhibitory activity [46]. Subsequently, six modified spongian diterpenes, ceylonins A–F, were isolated from another collection of the same sponge (Fig. 7). They contained an ether-bridged bicyclic ring system, which may be derived from spongia-13(16),14-dien-19-oic acid, a major metabolite of this sponge, and a C_3_ unit through intermolecular Diels–Alder reaction. Ceylonin A inhibited the formation of TRAP-positive multinuclear osteoclasts in RAW264 cells in a dose-dependent manner without cytotoxicity [47].

Aaptamine, bearing a 1*H*-benzo[*de*]-1,6-naphthyridine scaffold, was isolated from the marine sponge *Aaptos aaptos* collected in Okinawa, Japan, as an α-adrenoceptor blocking agent (Fig. 7) [48]. After its isolation, more than thirty congeners have been isolated from sponges thus far with differing biological activities. Among these, aaptamine, demethyl(oxy)aaptamine, and isoaaptamine inhibited RANKL-induced multinuclear osteoclast formation at 5 µM [49].

Seven triterpenes were isolated from the marine sponge *Siphonella siphonochalina* collected in the Red Sea. Among them, the pentacyclic neviotane-type triterpenes, neviotines A and D, inhibited multinuclear osteoclast formation with an IC_50_ value of 32.8 and 12.8 µM, respectively, whereas the IC_50_ values of other sipholane- and siphonellane-type triterpenes were higher than 50 µM (Fig. 7) [50].

Hymenialdisine is a bromopyrrole alkaloid isolated from the sponges *Axinella verrucosa* in the Mediterranean and *Acanthella aurantiaca* in the Red Sea (Fig. 7) [51]. This compound was selected from the library of marine natural products by screening of RANKL-induced osteoclastogenesis activity. It inhibited RANKL-induced osteoclast formation, bone resorption activity, and osteoclast-related gene expression by blocking the NF-*κ*B and MAPK signaling pathways and NFATc1 expression. Furthermore, hymenialdisine was suggested to induce osteoblast differentiation by activating alkaline phosphatase (ALP) and promoting osteoblast matrix mineralization. In addition, hymenialdisine prevented the decrease in bone volume and trabecular thickness in a female C57BL/6j mouse model of ovariectomy-induced systematic bone loss. Thus, hymenialdisine is a notable compound that both inhibits osteoclast-related osteolysis and promotes osteoblast-induced ossification, with in vivo efficacy [52].

Recently, a simple methylenedioxy dibromoindole alkaloid, amakusamine, was isolated from a marine sponge belonging to the genus *Psammocinia* collected in Amakusa, Kumamoto, Japan (Fig. 7). Amakusamine inhibited the formation of multinuclear osteoclasts in RANKL-stimulated RAW264 cells, with an IC_50_ value of 10.5 µM, via the suppression of *Nfatc1* expression. A series of amakusamine analogues was synthesized to examine their SAR, revealing that replacement of a methylenedioxy group with two methoxy groups slightly promotes activity. Hydrogenation of the Δ^2^ double bond reduced the activity. A bromine at C-4 is essential and bromination at C-7 slightly promoted activity. Replacement of the bromines with chlorines significantly reduced activity. Evaluation of the potencies of *N*-acyl derivatives demonstrated that those with C_2_ − C_8_-alkyl chains were equipotent or slightly more potent, but those with a C_14_-alkyl chain and benzoyl derivative were inactive even at 50 µM [53].

## Marine natural products that induce osteoblast differentiation

Although the therapeutic agents for osteoporosis are expected to be developed on the basis of compounds that suppress osteoclast differentiation or promote osteoblast differentiation, the number of osteoblast differentiation promotors isolated from natural sources is less than that of osteoclast differentiation inhibitors. In addition to hymenialdisine described above, the following compounds have been reported thus far.

**Fig. 8:**
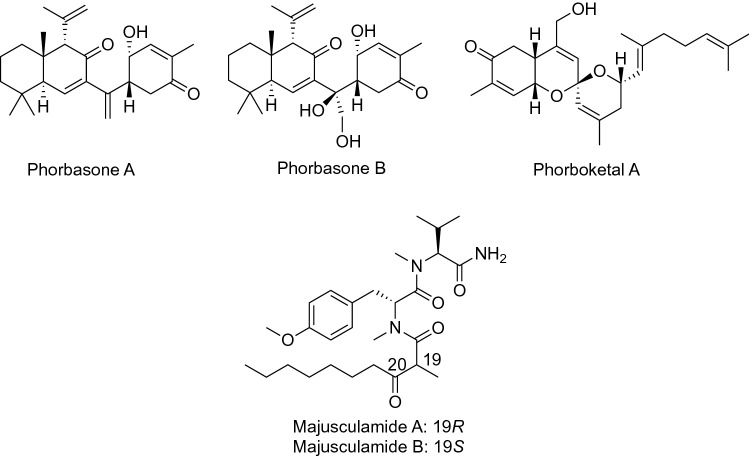
Structures of compounds that induced osteoblast differentiation

Phorbasones A and B, sesterterpenes, were isolated from the marine sponge *Phorbas* sp. collected at Gageo Island, Korea. Phorbasone A induced calcium deposition in mesenchymal C3H10T1/2 cells and the most potent effect was observed at 0.5 μg/mL (Fig. 8). Phorbasone A increased gene expression of the osteoblast differentiation markers, Runx2, ALP, OSX (osterix), PTH (parathyroid hormone), and PTHrP (PTH-related peptide) [54]. Furthermore, the same group screened a library of marine natural products and found that phorboketal A, previously isolated from the marine sponge *Phorbas* sp. collected at Gageo Island [55], promoted osteoblast differentiation in a concentration-dependent manner via ERK activation (Fig. 8) [56].

Majusculamides A and B are C-19 epimeric lipodipeptides isolated as the major products of the marine cyanobacterium *Lyngbya majuscula* Gomont collected at Kahala Beach, Oahu, Hawaii (Fig. 8) [57]. The same compounds were reisolated from the cyanobacterium *Moorea producens* collected at Bise, Okinawa, Japan, and induced osteoblast differentiation in MC3T3-E1 cells [58]. As majusculamide A was more potent than majusculamide B, the authors synthesized the analogues to assess SAR, and found that the numbers of methyl groups, configuration at C-19, and the functional groups at C-20 affected the activity. However, the carbon chain length of fatty acids and types of amino acid residues slightly affected the level of mineralization.

## Conclusion

More than half of the small molecules approved as drugs are either natural products or those derived from a natural product or based on a natural product pharmacophore [59]. Among natural products reported thus far, those discovered from marine environment comprise diverse chemical scaffolds accompanied by potent biological activities [16]. We reviewed marine natural products that inhibit osteoclastogenesis or promote osteoblast differentiation. Continuous effort may result in the discovery of drug leads for the treatment of osteoporosis.
